# Central bank digital currency, loan supply, and bank failure risk: a microeconomic approach

**DOI:** 10.1186/s40854-021-00296-4

**Published:** 2021-12-06

**Authors:** Jooyong Jun, Eunjung Yeo

**Affiliations:** 1 Department of Economics, Dongguk University, Seoul, 04620 Korea; 2 School of Business Administration, College of Business and Economics, Chung-Ang University, Seoul, 06974 Korea

**Keywords:** Central bank digital currency, Bank failure risk, Loan supply, D02, E51, E58, G21

## Abstract

Central bank digital currencies (CBDCs), which are legal tenders in digital form, are expected to reduce currency issuance and circulation costs and broaden the scope of monetary policy. In addition, these currencies may also reduce consumers’ need for conventional demand deposits, which, in turn, increases banks’ loan provision costs because deposits require higher rates of return. We use a microeconomic banking model to investigate the effects of introducing an economy-wide, account-type CBDC on a bank’s loan supply and its failure risk. Given that a CBDC is expected to lower the cost of liquidity circulation and become a strong substitute for demand deposits, both the loan supply and the bank failure risk increase. These increases are countered by subsequent increases in the rates of return on term deposits and loans, which, in turn, reduce the loan supply and thus bank failure risk. These offsetting forces lead to no significant change in banking, as long as the rate of return on loans is below a certain threshold. However, once the rate is above the threshold, bank failure risk increases, thereby undermining banking stability. The problem is more pronounced when the degree of pass-through of funding costs to the loan rate is high and the profitability of a successful project is low. Our results imply that central banks wishing to introduce an economy-wide, account-type CBDC should first monitor yields on bank loans and consider policy measures that induce banks to maintain adequate liquidity reserve levels.

## Introduction

Cryptocurrencies, such as Bitcoin and Ethereum, have penetrated mainstream financial markets. Facebook’s digital currency project, Diem (formerly known as Libra), has recently received substantial hype and has caused some controversy. Given these changing circumstances, central banks worldwide have gradually started to consider introducing “official” digital currencies, typically referred to as central bank digital currencies (CBDCs).^1^

Offering official or public currencies in a digital form has some advantages. They incur substantially lower issuance and liquidity circulation costs,^2^ reflecting their potential to mitigate the shortcomings of current private cryptocurrencies such as Bitcoin while still taking advantage of the digital format.^3^ In addition, CBDCs can expand the scope of monetary policy (for example, Bank for International Settlements 2018; Bordo and Levin 2017; Barontini and Holden 2019) and may enable other policy measures. For example, CBDCs can deliver quicker monetary relief to narrower targets; the importance of such relief is evident after the COVID-19 pandemic (for example, Auer et al. 2020).

However, introducing a CBDC also creates significant challenges for the current banking system, which is founded on the systematic relationship between a central bank and commercial banks. In particular, if the digital currency is an account-type currency,^4^ the typical commercial bank business model, which combines deposit acceptance and credit provision, may no longer be sustainable. Specifically, for a central bank, a CBDC is mainly a substitute for conventional paper bills and coins. However, for (retail) depositors, CBDC can substitute for a checking account, which is the most common type of demand deposit and is mostly used as a payment vehicle (Chiu et al. 2019). Thus, the economy-wide adoption of a CBDC may significantly lower consumers’ need for demand deposits, as it can effectively provide all economic agents with their demand deposit accounts at the central bank.^5^

As indicated by related studies (e.g., Keister et al. 2019; Chiu et al. 2019), after introducing a CBDC, banks should increase the interest rate on savings (including wholesale) or demand deposits,^6^ or both. Additionally, banks should maintain a proper level of liquidity reserves to address liquidity shocks, which have been regarded as the leading cause of bank runs, as noted by Bryant (1980), Diamond and Dybvig (1983) (BDD hereafter), and Goldstein and Pauzner (2005). A CBDC may help address this problem by embracing interim liquidity needs and allowing banks to maintain lower (excess) liquidity reserves. However, banks should continue to maintain liquidity reserves and draw them down in times of stress, such as the global financial crisis in the late 2000s, because stress can trigger runs on their mostly unprotected term deposits. Reduced demand deposits can hamper banks’ ability to maintain proper levels of liquidity reserves.

We examine the effects of anticipated changes in the rates of return after an economy-wide, account-type CBDC is introduced by modifying and extending the global game bank run model of Freixas and Ma (2014).^7^ We specifically focus on the effects on a bank’s choice of loan supply and its failure risk. The following findings are obtained. First, the decrease in the currency circulation cost due to the introduction of a CBDC increases both the loan supply and bank failure risk. However, the increase in the rate of return on term deposits and a subsequent increase in the rate of return on loans have the opposite effect on loan supply. Subsequently, no significant change will occur in the loan supply. Meanwhile, two different outcomes are possible for the bank failure risk, depending on the profitability of borrowers, that is, the difference between the rate of return on a successful business and the rate of return on loans. If the borrowers’ profitability, which is inversely related to the borrower’s moral hazard problem, is sufficiently high, the bank failure risk decreases, countering the effect of the lower circulation cost of currency. The eventual bank failure risk does not change significantly, as observed in the case of loan supply. By contrast, if the borrowers’ profitability is low, the bank failure risk will *increase*, implying a significant adverse impact.

The intuition for these results is as follows. On the one hand, the introduction of a CBDC creates incentives for banks to hold lower (excess) liquidity reserves and subsequently increase their loan supplies. On the other hand, CBDC accounts attract some liquidity away from banks’ demand deposits and, in turn, decrease bank deposits for funds. In such a situation, the rate of return for deposits needs to increase, following the decrease in the supply of the loanable funds. Thus, no significant changes are likely to occur in both loan supply and bank failure risk. However, if the rate of return on loans is above a certain threshold, borrowers’ moral hazard problems materialize, and consequently, the default risk of loans increases, implying that the adverse effect of the increase in rates starts to dominate its benefit, that is, risk reduction occurs from an increase in banks’ liquidity buffer. Given the setting of the competitive deposits market on the one hand and the monopolistic loan market on the other, a high degree of pass-through of the increase in cost (deposit rate) from the deposit side to the loan side and subsequent undermining of bank stability are likely to occur. From a policy standpoint, to ensure banking prudence after the introduction of a CBDC, central banks should first monitor yields on bank loans and consider taking complementary policy measures to help banks maintain proper liquidity reserve levels if necessary.^8^

Several previous studies have examined the impacts of CBDC introductions from perspectives similar to ours. For instance, Parlour et al. (2020) investigate the payment system innovation effects of CBDC introductions without considering bank failure risk and conclude that introducing a CBDC increases liquidity needs and decreases economic output. Keister et al. (2019) and Kim and Kwon (2019) find that although a CBDC tends to promote exchange efficiency, it can crowd out bank deposits, raise banks’ funding costs, and decrease investment. Kim and Kwon (2019) further conclude that a CBDC has negative effects on financial stability. By contrast, Chiu et al. (2019) suggest that a CBDC can improve lending because it serves as a viable outside option for bank deposits and thus disciplines oligopolistic banks’ behavior by changing the interest rate. Fernández-Villaverde et al. (2020) suggest that a CBDC helps avoid bank runs because the central bank becomes a rigid, monopolistic depositor. Brunnermeier and Niepelt (2019) also conclude that CBDC introductions are neutral to credit provision and bank risk because public and private money are interchangeable via monetary policy. Adopting monopolistic banking, Andolfatto (2021) finds that the presence of CBDC would increase the cost of deposit funding for the bank. However, its monopoly status can help it partially absorb the increase in funding costs and even expand bank lending. Although many features of the setting are common to ours (e.g., pass-through of rates), he mainly focuses on changes in banking activities, whereas we mainly focus on bank failure risk.

Our main contribution to the literature is identifying conditions under which adverse effects on bank failure risk are more pronounced with the presence of an account-type CBDC. The degree of pass-through of the increase in deposit rate to the loan market and the borrowers’ moral hazard jointly affect the rate of return on loans and thus the riskiness of loans and banks’ profits. Although competition in the deposit market leads to a high degree of pass-through of deposit rates to the loan market, banks’ market power in the loan market can help absorb some of the cost increase. If the offsetting effects eventually lead to a sufficiently high rate of return on loans, which is more likely if loan demand is inelastic, bank failure risk will subsequently increase.

Since the work of BDD, theoretical studies on banking have focused on the flow of liquidity in the money-credit system and bank failure risk due to a bank run.^9^ To the best of our knowledge, this study is the first in this strand of the literature to implement a full theoretical analysis of the effects of CBDC presence. The remainder of this paper is organized as follows. “Model” section models a bank’s portfolio, defined as liquidity reserves and loan supply, in addition to (in)solvency and (il)liquidity risks, the two major bank risks associated with portfolio choice. “Equilibrium following the introduction of a CBDC” section provides the results in two possible scenarios and their implications based on the comparative statics of the key variables. Finally, “Discussion and conclusion” section concludes the paper and provides policy suggestions and directions for future work.

## Model

We adapt Freixas and Ma (2014)’s global game bank-run model by incorporating the factors affecting a bank’s chosen liquidity reserves level and loan supply when a CBDC is introduced.

### Setting

Two regions, denoted by $i \in \{1, 2\}$, were used in this study. Each region has one bank, also denoted by *i*.^10^ A borrower in region *i* can obtain a loan only from bank *i*. However, we assume that bank *i*, monopolistic in the loan market of region *i* (hereafter referred to as loan market *i*), cannot completely control the gross rate of return on loans, denoted by $R_i(>1)$, and we treat it as an exogenous variable. This assumption is based on the fact that interest rates are typically affected by other factors such as borrowers’ bargaining power and regulatory interventions, although a bank can reflect, or pass through, a change in the rate of return on deposits upon the rate of return on loans. By contrast, we assume a competitive deposit market wherein banks make zero profit. Depositors in either region can choose a bank in either region, creating the need for an interbank settlement and clearing service. In our setting, the system does not seek excess profits for this service. That is, if the operating costs are low (high), banks are charged a low (high) unit cost, denoted by $\tau$, for interbank payment and settlement.

We show the T-account of bank *i* in Table 1. The liability side of bank *i*’s portfolio is composed of demand deposits, denoted by $F_i$, and term deposits,^11^ denoted by $D_i$. The asset side is composed of bank *i*’s loan supply, denoted by $d_i$, which is the only choice variable of the bank and refers to the *fraction* of all applicants who receive a loan from bank *i*, and the level of liquidity reserve, which is denoted by $f_i$. Note that $d_i$ reflects the (normalized) loan supply; thus, $f_i$ is determined accordingly. In other words, $f_i + d_i$ is not necessarily equal to 1. We normalize the values so that $F_i + D_i=f_i + d_i$.^12^ Finally, 1/*e* refers to an exogenous reserve ratio for demand deposits, which leads to $F_i = ef_i$.

**Table 1:** Bank’s T-account

Assets	Liabilities
Loan ($d_i$)	Term deposits ($D_i$)
Liquidity reserve ($f_i$)	Demand deposits ($F_i$)

Let $R_c(\ge 1)$ and $R(\ge 1)$ denote the gross rates of return on demand deposits and term deposits, respectively. We assume that $R_c$ and *R* are exogenous and similar in both regions, reflecting the competitiveness of the deposit market. We also assume that $R/(R-1)>e$, which excludes the case in which a bank provides an unreasonably high rate of return on term deposits for a given reserve ratio. The average gross rate of return on an entrepreneur’s successful project in region *i* is denoted by $X_i$.

Note that $X_i - R_i$ is a borrower’s profitability from a successful project. Following Freixas and Ma (2014), we assume that $X_i - R_i$ affects borrowers’ moral hazard: the smaller the value of $X_i - R_i$, the lower the level of effort and the higher the default risk of loan. Following Freixas and Ma (2014), let $b \in (0, B_i]$ denote a borrower’s type, with a higher *b* implying a better (less risky) type, and is uniformly distributed along the interval $(0, B_i]$. Thus, a higher value of $B_i$ implies a greater number or the proportion of safe borrowers. We also assume that information asymmetry about types does not exist, and the moral hazard problem worsens for a lower value of *b*. Therefore, bank *i* will provide loans first to the highest/safest types and, as the loan supply $d_i$ increases, the lower/riskier types will obtain their loans. Adapting the result derived from Freixas and Ma (2014),^13^ we define the fraction of riskless loans, ${\alpha _i}$, as

$$\begin{aligned} \alpha _i = 1-d_i/(B_i(X_i - R_i)), \end{aligned}$$

where $B_i(X_i-R_i)$ can be interpreted as $B_i$ *after borrowers’ moral hazard is adjusted*.

We assume that $X_1 < X_2$ and $B_1 > B_2$, implying that the average business project is a low-risk and low-return project in region 1, whereas it is a high-risk and high-return project in region 2. We also assume that $B_1(X_1 - R_1) > B_2(X_2 - R_2$). As $d_i$ and $R_i$ increase, $\alpha _i$ decreases. Note that a lower $\alpha _i$ is associated with a higher failure risk for bank *i*.

The cash flow $\theta _i$ generated for bank *i* from a *unit* loan is

1 $$\begin{aligned} \theta _i&= \alpha _i R_i + (1-\alpha _i)[0 \cdot \gamma _i + R_i \cdot (1-\gamma _i)] \nonumber \\&= R_i - (1-\alpha _i)R_i \gamma _i = R_i \left( 1- d_i \gamma _i /(B_i(X_i-R_i))\right) , \end{aligned}$$

where $\gamma _i$ is the probability of loss on a risky loan (i.e., credit risk) in loan market *i*, which is assumed to follow a uniform probability distribution on (0, 1).

### Deposits run game

The deposit run game uses a three-stage structure ($t=0,1,2$) that has been adopted by many similar studies since the BDD. The game proceeds as follows.

#### (1) $t=0$

Bank *i* constructs its asset portfolio ($f_i,d_i$). The level of liquidity reserves $f_i$ is directly linked to the amount of demand deposits $F_i$, implying that the bank only uses demand deposits for liquidity reserves.

#### (2) $t=1$

Term (and wholesale) depositors can choose to withdraw their deposits early by forgoing their returns at $t=2$.^14^ The proportion of this depositor type is denoted by $L_i$($0< L_i < 1$) for bank *i*. Unlike BDD, we assume no net outflow of demand deposits, which are protected by deposit insurance.^15^ Thus, the decision to liquidate early is based solely on depositors’ beliefs about whether the bank is likely to be solvent at $t = 2$, rather than on their actual liquidity needs. This implies the possibility of speculative runs on term and wholesale deposits (e.g., Rochet and Vives 2004), which are less liquid and thus less likely to be convertible to public liquidity. Depositor *n* makes an early withdrawal decision based on her private signal $s_n^i = \theta _i + \epsilon _n^i$ about the bank’s cash flow, where $\epsilon _n^i$ is a small, non-zero error that is independent and identically distributed.

If a bank has little liquidity to meet the demand for early withdrawals, illiquidity problems arise, regardless of whether the bank would remain solvent at $t=2$. Thus, a bank must be able to either secure liquidity at $t = 0$, borrow liquidity from the repo market or other interbank markets, or sell its financial claims in the money market at $t=1$. We denote the average gross rate of the *discount* for the interim liquidation of a bank’s financial assets as $R_b$.^16^

#### (3) $t=2$

Through the settlement and clearing process, successful borrowers repay their loans, and solvent banks return their deposits and returns to depositors. If bank *i* is solvent, it returns $R(1-L_i)d_i$ to an owner of term deposits who did not choose to withdraw at $t=1$. However, if a bank failure occurs, unprotected depositors would receive nothing. As in the setting of Parlour et al. (2020), we assume that banks incur a transfer cost that is proportional to the net liquidity outflow for the use of the interbank settlement and clearing process,^17^ denoted by $\tau \cdot \max \{0, d_i - d_j\}$ or $\tau (d_i - d_j)^+$, if necessary. This implies that even if a bank’s net liquidity outflow is less than zero, it receives no extra income.

We assume the following relationships between the major variables:

(i) $$0<\tau< f_i \ll d_i<1 \ll e < R/(R-1)$$;
(ii) $$1<R_c< R< R_b< R_i < X_i$$;
(iii) $$R_b>R+\tau$$;
(iv) $$B_i(X_i-R_i) > 2$$;
(v) $$R_b < 1+\tau R_i/(2f_i)$$;

Assumption (i) is necessary to obtain an interior solution and is not significantly different from reality.^18^ Assumption (ii) is also based on observable rates in practice. Assumption (iii) implies that if relatively less liquidity is secured in advance and if many early withdrawal requests are made, the cost of financing the necessary liquidity is greater than the cost of financing saving deposits, although it is not too much greater. Assumption (iv) implies that at least half of the loans are riskless, even if all applicants receive loans, implying that $d_i > 0.5$. Finally, Assumption (v) is a sufficient condition that guarantees the existence of an interior solution of $d_i \in (0,1)$, which is used in “Bank’s loan supply choice” section.

### Bank failure risk

Although banks face various kinds of risks that can cause bank failure, this study focuses on two major bank risks that are usually regarded as the main causes of individual bank failures: (in)solvency risk and (il)liquidity risk.

#### Insolvency risk

A bank is *solvent* if the cash flow generated from its loans is greater than the sum of its deposits and the interest that it owes.^19^ That is,

2 $$\begin{aligned} d_i\theta _i = d_i\left( R_i - (1-\alpha _i) R_i \gamma _i \right) \ge R_c F_i + R D_i + \tau (d_i - d_j)^+ = \phi _i +Rd_i + \tau (d_i - d_j)^+ \end{aligned}$$

must be satisfied, where $\phi _i = (R - Re +R_c e)f_i$, which is positive and less than $f_i$ because $R/(R-1) > e$ in Assumption (i). We conjecture that $\tau$ is insignificant in this case. From Eq. (2), we define the critical level of the probability of a loss on a risky loan, henceforth the *critical loss level*, in region *i* as

3 $$\begin{aligned} {\bar{\gamma }}_i = ( R_i - (\phi _i/d_i + R + \tau )) /((1-\alpha _i) R_i). \end{aligned}$$

Bank *i* remains solvent if $\gamma _i$ is less than ${\bar{\gamma }}_i$. Because $\gamma _i$ is assumed to follow a uniform distribution *U*[0, 1], the probability that bank *i* is solvent is $\Pr (\gamma _i < {\bar{\gamma }}_i) = {\bar{\gamma }}_i$. Thus, the (in)solvency risk of bank *i*, $\rho ^{SR}_i$, is determined as follows:

4 $$\begin{aligned} \rho ^{SR}_i \equiv 1-{\bar{\gamma }}_i = (\phi _i /d_i +R+\tau - \alpha _i R_i)/((1-\alpha _i) R_i). \end{aligned}$$

In addition to the (in)solvency risk, *(il)liquidity risk* must also be considered. As mentioned above, early withdrawal attempts or runs by depositors can occur based on the *possibility* of a bank failure in our model.^20^ If the inequality

$$\begin{aligned} \theta _i / R_b > 1 \end{aligned}$$

is satisfied, the bank has no liquidity problem at $t=1$. If the inequality

$$\begin{aligned} (1-L_i)(d_i (R+\tau ))+ \phi _i&\le ( \theta _i - L_iR_b)d_i \end{aligned}$$

is also satisfied at $t=2$, the bank is solvent. The proportion of early withdrawals at which no bank failure occurs at $t=2$, denoted by $L_i$, must satisfy

5 $$\begin{aligned} L_i \le L_i^* = \frac{\theta _i - (\phi _i/d_i + R+\tau ) }{R_b - (R+\tau ) }, \end{aligned}$$

where $L_i^*$ is the threshold early withdrawal proportion level below which bank *i* is solvent. Clearly, the higher the cash flow level, the higher the value of $L_i^*$.

#### (Il)liquidity risk

To focus on the (il)liquidity risk, we suppose that no bank failure occurs at $t=1$. In the worst case, in which only risk-free loans are repaid, each bank’s cash flow from a unit loan $\theta _i = \alpha _i R_i$ must satisfy $\alpha _i R_i > R_b$ for the bank to stay solvent.

Depositor *n*’s early withdrawal decision is purely speculative and determined by her belief about other depositors’ decisions given her private signal $s^i_n = \theta _i + \epsilon _n^i$. Note that common knowledge is not established in this setting because we cannot specify in advance the proportion of depositors who choose to run, denoted by $L_i$. A depositor’s choice is influenced by other depositors’ *beliefs* about $L_i$ after observing $s^i_n$ rather than by $L_i$.

We use a model of a binary-action global game with the Laplacian property in which depositor *n* chooses a switching strategy; she withdraws early if $s_n^i$ is lower than the threshold $s^*$ in equilibrium. She believes that the aggregate action of other depositors, denoted by $a \in [0,1]$, is $a = 1-\Pr (s_n^i>s^*)$, which should be equal to the mass of agents with noise above her own $\epsilon _n^i$ such that $a = 1- H(\epsilon _n^i)$, where *H*() is the probability distribution. Note that she does not observe the noise $\epsilon _n^i$; thus, she regards $H(\epsilon _n^i)$ and subsequently *a* as random variables from a uniform distribution, known as the Laplacian property^21^ in global games. Morris and Shin (2001) show that if the Laplacian property is satisfied, a unique switching strategy equilibrium exists in a binary-action global game.

##### **Lemma 1**

*There exists a range of cash flows that can cause illiquidity and a run on bank i, even if it is immune to insolvency if the cash flow falls within the range of*

6 $$\begin{aligned} \phi _i /d_i + R + \tau< \theta _i < \phi _i/d_i + \left( R+\tau + R_b / (R+\tau ) -1\right) = \phi _i/ d_i + \mu \end{aligned}$$

*where*

7 $$\begin{aligned} \mu = R+\tau + R_b/(R + \tau ) -1 > R+\tau . \end{aligned}$$

##### *Proof*

See “Appendix 2”. $$\square$$

Given $\theta _i \sim U[\alpha _i R_i, R_i]$ and Eq. (6), the likelihood that bank *i* experiences illiquidity risk despite being immune to the solvency problem is expressed as follows:

8 $$\begin{aligned} \rho ^{IR}_i \equiv (\mu - (R+\tau ))/((1-\alpha _i) R_i). \end{aligned}$$

#### Bank failure risk

Finally, the *bank failure risk* of bank *i*, denoted by $\rho _i$, is defined as the combination of its solvency and liquidity risk, that is, the sum of Eqs. (4) and (8), as given below:

9 $$\begin{aligned} \rho _i = \frac{\phi _i /d_i + \mu - \alpha _i R_i}{(1-\alpha _i) R_i} = 1-(B_i(X_i - R_i))( R_i - (\phi _i /d_i + \mu ))/(d_iR_i). \end{aligned}$$

From Eq. (9), bank *i*’s critical loss level in incorporating the (il)liquidity risk, denoted by ${\hat{\gamma }}_i$, is derived as

10 $$\begin{aligned} {\hat{\gamma _i}} =1-\rho _i&= ( R_i - (\phi _i /d_i + \mu ))/((1-\alpha _i) R_i)\text{, } \text{ or } \nonumber \\&= B_i(X_i - R_i)( R_i - (\phi _i /d_i + \mu ))/(d_iR_i). \end{aligned}$$

As the loan amount increases, the critical loss level decreases, and the bank failure risk increases. The first derivative of ${\hat{\gamma _i}}$ with respect to $d_i$ is

$$\begin{aligned} \frac{\partial {{\hat{\gamma }}}_i }{\partial d_i }=-\frac{B_i(X_i - R_i)(R_i-2\phi _i/d_i - \mu )}{d_i^2R_i}<0, \end{aligned}$$

assuming that $R_i$ is sufficiently greater than *R* (i.e., $R_i > 2\phi _i/d_i + \mu$), implying that the bank failure risk $\rho _i$ increases with respect to $d_i$.^22^

### Bank’s loan supply choice

Bank *i*’s expected profit from loan supply $d_i$ can be represented as follows:

$$\begin{aligned} E_{\gamma _i}[ \pi _i ]&=E_{\gamma _i} [ \theta _i d_i-(f_i+R d_i) - \tau (d_i - d_j)^+ ] \\&=E_{\gamma _i} [ (R_i - (1-\alpha _i)R_i \gamma _i) d_i -f_i -R d_i- \tau (d_i - d_j)^+] \\&\text{ s.t. } 0< d_i < 1. \end{aligned}$$

Although bank failure risk is an important factor to consider from the perspective of financial stability, managers’ decisions are often subject to limited liability. That is, when a bank fails, its managers may receive no performance-based compensation, but this is their only loss.

For many borrowers, we can regard the critical loss level ${\hat{\gamma _i}}$ as the *proportion* of non-performing loans. Thus, when a bank’s managers choose the loan supply $d_i$ to maximize the bank’s expected payoff under limited liability, they treat the case $\gamma _i > {\hat{\gamma _i}}$ as if the net cash flow between loans and term deposits is zero and exclude it from their consideration.

Note that in the case of bank failure, bank *i* returns its liquidity reserves $f_i$, rather than $\phi _i$, to the deposit insurance agency, which initially repays the protected deposits to the depositors if a bank failure occurs. In addition, many payment systems require banks to *pre*-deposit collateral proportional to net liquidity outflows, and the bank is unlikely to regain this collateral if a bank failure occurs. The bank’s expected profit can therefore be represented as

11 $$\begin{aligned} E[\pi _i]&= \int _0^{{{\hat{\gamma }}}_i} [ (R_i - (1-\alpha _i)R_i \gamma )d_i -Rd_i] d\gamma -\tau (d_i - d_j )^+ - f_i \nonumber \\ =&\frac{B_i(X_i - R_i)}{R_i}\left[ -(R_i - (\phi _i /d_i + \mu ))^2/2 +(R_i - R)(R_i - (\phi _i /d_i + \mu )) \right] - \tau (d_i - d_j )^+ -f_i. \end{aligned}$$

The bank’s problem, or, more precisely, the manager’s problem is to choose the value of $d_i$ that maximizes the expected profit under managers’ limited liability in the case of bank failure. Let $Z = R_i - (\phi _i /d_i+\mu )$. Then, Eq. (11) becomes

$$\begin{aligned} \frac{B_i(X_i-R_i)}{R_i} \left( -Z^2/2 + (R_i-R)Z \right) -\tau (d_i-d_j)^+-f_i, \end{aligned}$$

which is a quadratic function of *Z*. We can now find the profit-maximizing (normalized) loan amount from the first-order condition with respect to *Z*, which is

$$\begin{aligned} \frac{B_i(X_i-R_i)}{R_i} \left( -Z + R_i-R \right) \frac{f_i}{d_i^2}-\tau =0. \end{aligned}$$

Knowing that $-Z+R_i-R = \phi _i/d_i + \tau + R_b/(R+\tau )-1$, this equation leads to

12 $$\begin{aligned} \phi _i +(\tau + R_b/(R+\tau ) -1)d_i - \frac{\tau R_i}{B_i(X_i-R_i)}\frac{d_i^3}{f_i} =0. \end{aligned}$$

#### **Lemma 2**

*A unique interior solution exists for bank i’s choice of loan supply* $d^*_i \in (0,1)$.

#### *Proof*

The left-hand side of Eq. (12) is $\phi _i$, which is a minuscule, but still positive when $d_i=0$. Assumptions (i), (iv), and (v) imply that the sign of Eq. (12) is negative, or

$$\begin{aligned} \phi _i +(\tau + R_b/(R+\tau ) -1) - \frac{\tau R_i}{B_i(X_i-R_i)}\frac{1}{f_i}< \phi _i + (R_b -1) - \tau R_i /(2f_i)<0 \end{aligned}$$

when $d_i=1$. Next, given that Eq. (12) is a cubic function with no second-order term and a negative third-order term, there exists a unique interior solution $d^*_i \in (0,1)$. $\square$

Although deriving the exact form of $d_i$ from Eq. (12) is impractical, Lemma 2 implies that we can still perform comparative statics and analytically derive the direction of changes. First, note that $\partial d^*_i/ \partial \phi _i >0$. Thus, if necessary, we can use an approximate but closed-form solution for our comparative statics by removing $\phi _i=(R-Re+R_ce)f_i$ from the left-hand side of Eq. (12), reflecting $\phi _i \ll d_i$, as

13 $$\begin{aligned} d_i^2 \approx \frac{B_i(X_i-R_i)f_i}{\tau R_i} \left( \tau + R_b/(R+\tau ) -1 \right) . \end{aligned}$$

Thus, if the signs of the comparative statics results for $d^*_i$ and $\phi _i$ are the same, we can safely use $d_i$ from Eq. (13) for comparative statics.

“Appendix 1” provides comparative static results for ${\hat{\gamma }}_i$ and $d_i$ derived from Eq. (13) for the major variables.

## Equilibrium following the introduction of a CBDC

Successful adoption of a CBDC will alter consumers’ preferences for the types of liquidity—cash, checking account, and CBDC account. We expect that the introduction of a CBDC with economy-wide accessibility makes CBDC the primary type of liquidity and has the following two effects. First, the CBCD would reduce the need to handle conventional cash and, therefore, significantly lower the cost of issuing and circulating currency.^23^ Second, the CBDC also attracts a significant amount of liquidity, transactional deposits, for example, beyond conventional demand deposits, which banks use to finance their loan supplies and maintain their liquidity reserves at lower costs, compared with term deposits. We now check the subsequent changes in the loan supply and bank failure risk.

Suppose that CBDCs and demand deposits are strong substitutes and that a significant portion of demand deposits switches to the CBDC account.^24^ From a bank’s perspective, the amount of total deposits that can be used to fund loans decrease. In addition, banks’ ability to secure liquidity reserves is constrained because bank *i*’s (normalized) liquidity reserve $f_i=F_i/e$ is tightly linked to the amount of demand deposits $F_i$.

Let the utility of depositor *n*, who has a demand deposit account at bank *i*, be

$$\begin{aligned} u_i(l_n) + R_cl_n + R\sigma _n, \end{aligned}$$

where $l_n$ is the amount of demand deposits, $\sigma _n$ is the amount of term deposits, and $u_i()$ is the increasing concave non-monetary utility from holding demand deposits, such as a checking account, with bank *i*. Let $w_n=l_n+\sigma _n$ be depositor *n*’s initial liquidity endowment. Then, the first-order condition is

14 $$\begin{aligned} u'_i(l_n) = R - R_c, \end{aligned}$$

implying that a marginal change in either *R* or $R_c$ changes the marginal utility of $l_n$ by the same degree but in the opposite direction.

### **Proposition 1**

*After the introduction of an account-type CBDC, the rate of return for term deposits increases.*

### *Proof*

From the comparative statics results, we know that $\partial d^*_i/\partial R_c >0$ and $\partial d^*_i / \partial R <0$. As long as the initial liquidity endowment $w_n$ does not change, the introduction of a CBDC first reduces banks’ total amount of conventional demand deposits and loanable funds. Consequently, the equilibrium loan supply, $d^*_i$, decreases, implying an eventual increase in *R*. $\square$

When the rate of return on deposits increases in the demand deposits market, assumed to be competitive, after the introduction of a CBDC, we should consider the pass-through of the cost increase to the loan market because banks make no profit in the deposit market, but they are monopolistic in the loan market. That is, the exogenous change in the cost—the rate of return for deposits—is reflected in its price—the rate of return on loans. We provide some intuition here.

First, note that $d_i$ is not affected by any feature related to borrowers’ demand. That is, every potential borrower wants to obtain a loan as long as $X_i - R_i > 0$, implying an inelastic demand for loans in equilibrium. Because the deposit market is competitive, the increase in the funding cost (deposit rates) after the CBDC introduction is not absorbed in the deposit market, and a significantly high degree of, if not perfect, pass-through of the cost to the monopolistic loan market will occur unless the increase in $R_i$ can be curtailed by other external forces.

For the sake of comparison, let us assume away the possibility of any interest rate pass-through. Then, after the introduction of the CBDC, changes in banks’ loan supplies and bank failure risk will be insignificant, and their directions will be indeterminate. From “Appendix 1”, we know that $\partial d_i/\partial R <0$, $\partial d_i/\partial \tau <0$, $\partial {\hat{\gamma _i}}/\partial R > 0$, and $\partial {\hat{\gamma _i}}/\partial \tau > 0$. The bank loan supply, $d_i$, decreases, and the bank failure risk, $\rho _i = 1- {\hat{\gamma }}_i$, decreases as *R* increases. However, these effects are somewhat offset by the reduced value of $\tau$. Although the direction of the aggregate effect is uncertain, we can conclude that the changes in $d_i$ and $\rho _i$ are not significantly large.

Now, we consider the pass-through of the increase in the rate of return on deposits to the rate of return on loans, by which an increase in *R* leads to a consequential increase in $R_i$. We find that the effect of the introduction of an account-type CBDC after considering the cost pass-through varies depending on the characteristics of the loan market. Unlike the rest of the comparative statics results, the sign of $\partial {\hat{\gamma _i}} / \partial R_i$ is indeterminate and is affected by the value of $X_i - R_i$.

As mentioned previously, following Freixas and Ma (2014), we assume that a high $X_i - R_i$ reduces borrowers’ moral hazard; thus, bank *i* is willing to underwrite loans for the lower rate of return on loans $R_i$. Hence, in addition to measuring borrower profitability, we provide another interpretation of $X_i - R_i$, as shown in the following definition.

### **Definition 1**

Loan market *i* is considered *more favorable* for borrowers as $X_i - R_i$ *increases*.

**Fig. 1:**
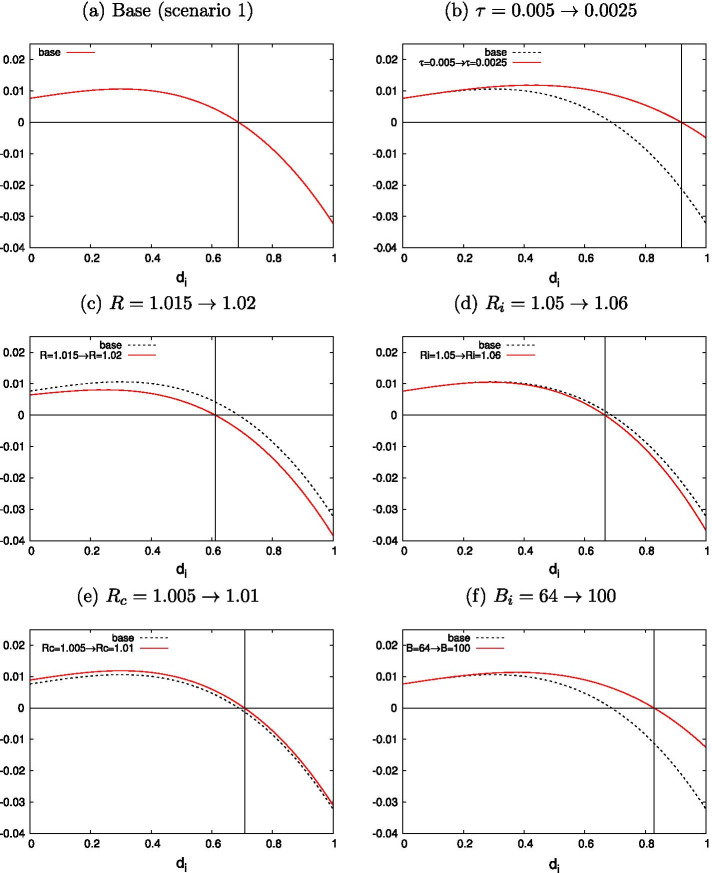
Comparative static: a numerical example (Scenario 1)

**Fig. 2:**
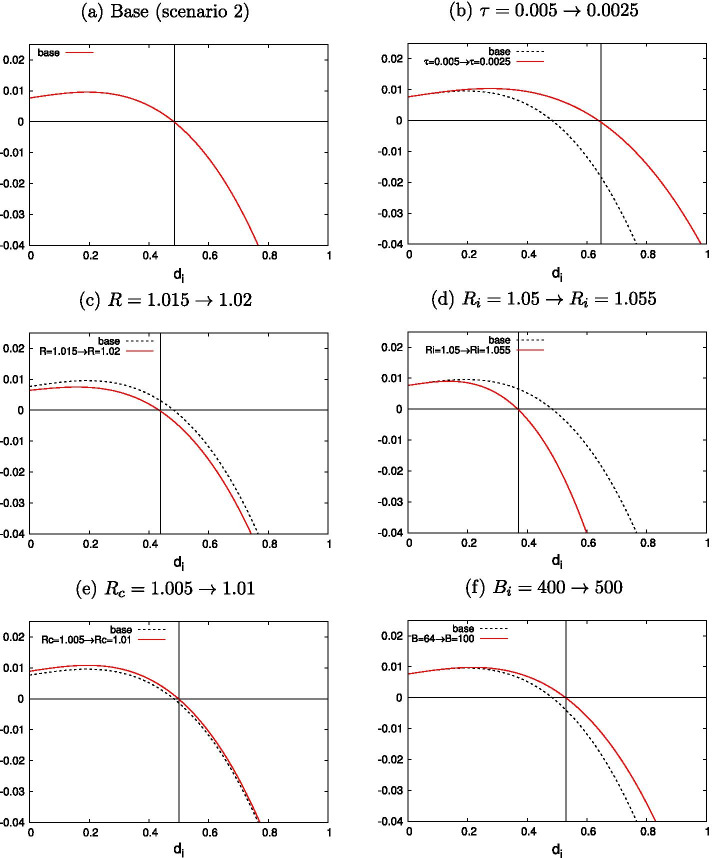
Comparative static: a numerical example (Scenario 2)

In the first scenario, the loan market is more favorable to borrowers, meaning that $X_i - R_i$ is large enough to satisfy $\partial {\hat{\gamma _i}} / \partial R_i > 0$. In the second scenario, the loan market is less favorable to borrowers, meaning that $X_i - R_i$ is small enough to satisfy $\partial {\hat{\gamma _i}} / \partial R_i < 0$. Figures 1 and 2 show numerical examples of the interior solution $d_i^*$ and its changes concerning each major variable under the first and second scenarios, respectively, without using the approximate closed-form solution of Eq. (13).

Each subfigure in Fig. 1 provides a numerical description of the comparative static result for the first scenario. Figure 1a describes the base for our analysis, with an interior solution of $d^*_i = 0.688$, given that $\tau = 0.005$, $R=1.015$, $R_c=1.005$, $R_b=1.03$, $R_i=1.05$, $f_i = 0.01$, $e=25$, $B_i=64$, and $X_i=1.2$. Here, $X_i - R_i = 0.15$ is sufficiently high. We use these values as our base.^25^ We can observe that the directions of the changes in $d_i$ are the same as those described in “Appendix 1”. Figure 1d shows the change in $d_i$ when $R_i$ increases by 0.01 (1%) from the base case described in Fig. 1a. ${\hat{\gamma _i}}$ increases from 0.180 to 0.316.

Meanwhile, each subfigure in Fig. 2 describes the numerical comparative static results for the second scenario. Some of the base parameters are changed; specifically, $B_i = 400$ and $X_i = 1.06$, which leads to $d^*_i=0.480$, as shown in Fig. 2a. Note that $X_i - R_i = 0.01$ is far smaller than the value from the first scenario, and if $R_i$ increases by 0.005, as in Fig. 2d, ${\hat{\gamma _i}}$ now *decreases* from 0.0356 to 0.0238.

Using the comparative static results for the (normalized) amount of loan and the critical loss level, described in “Appendix 1: Comparative statics” section, we establish Proposition 2, which states the effects of the introduction of a CBDC on a bank’s loan supply and its failure risk in each scenario.

### **Proposition 2**

*Given the pass-through of the cost (deposit rate) increase to the price (rate of return on loan), the introduction of an economy-wide, account-type CBDC leads to the following outcomes, depending on the borrower’s profitability in the loan market proxied by* $X_i - R_i$:

1. In the first scenario, in which the loan market is sufficiently more favorable for borrowers (i.e., $X_i - R_i$ *is sufficiently large), the overall change in the loan supply and the bank failure risk is not significant.*
2. *In the second scenario, in which the loan market is sufficiently less favorable for borrowers (i.e.,* $X_i - R_i$ *is sufficiently small), the overall change in the loan supply is not significant either. However, the risk of bank failure increases.*

### *Proof*

Note first that the introduction of a CDBC should decrease the value of $\tau$. $\partial d_i/\partial \tau <0$ and $\partial {\hat{\gamma _i}}/\partial \tau >0$, taken from “Appendix 1”, imply that the (normalized) loan supply $d_i$ and the bank failure risk $\rho _i$ increase after the introduction of a CBDC.

Meanwhile, $\partial d_i/\partial R <0$ and $\partial d_i/\partial R_i <0$, taken from “Appendix 1” imply that the increase in *R* and the subsequent increase in $R_i$ because of the pass-through of the deposit rate to the loan rate unambiguously decreases the (normalized) loan supply ($d_i$), which is opposite to the effect of the decrease in $\tau$. Therefore, we can conclude that the change in $d_i$ is not significant.

The effect on $\rho _i$ has two scenarios. In the first scenario, in which $X_i - R_i$ is large, both $\partial {\hat{\gamma _i}}/\partial R >0$ and $\partial {\hat{\gamma _i}}/\partial R_i >0$ are positive, implying that both the loan supply and bank failure risk decrease and that the overall changes in $d_i$ and $\rho _i$ are not significant. Conversely, in the second scenario, in which $X_i - R_i$ is small, $\partial {\hat{\gamma _i}}/\partial R >0$ and $\partial {\hat{\gamma _i}}/\partial R_i <0$ have the opposite signs. Thus, for a sufficiently small $X_i - R_i$, the (normalized) loan supply ($d_i$) decreases, implying that the effect of the lower $\tau _i$ and that of the higher $R_i$ counter against each other. In contrast, the risk of bank failure ($\rho _i$) increases owing to both a lower $\tau _i$ and a higher $R_i$.$\square$

In the first scenario, the risk reduction from the reduced loan supply dominates the increased default risk of borrowers, thereby decreasing the bank failure risk. By contrast, in the second scenario, the increase in borrowers’ default risk due to the increase in $R_i$ may be greater than the reduced risk of the lowered supply of loans to borrowers with relatively lower credit ratings. Given that liquidity circulation cost decreases with the introduction of the CBDC, no significant change in the loan supply or bank failure risk would occur in the first scenario. However, the bank failure risk ultimately increases without any significant change in the loan supply in the second scenario. Combined with the assumptions regarding riskiness and the rates of return in each market, $B_1(X_1-R_1) > B_2(X_2-R_2)$ and $X_1 - R_1 < X_2 - R_2$. Proposition 2 implies that market 1 is more likely to have an adverse effect on bank failure risk after the introduction of a CBDC. This result also implies that introducing competition in the loan market would be helpful to reduce the bank failure risk specifically if the loan demand is highly inelastic.

We briefly discuss the effect of bank capital requirement. Suppose that the cost of capital is higher than the cost of debt due to the equity premium, and the bank maintains the minimum required ratio of capital. That is, the average cost of raising funds for loans is $R(1-\kappa )+RK\kappa > R$, where $\kappa <1$ is the ratio of equity to the entire loan from unprotected sources, and $RK (K>1)$ reflects the equity premium. Thus, the amount of term deposit *as the bank’s liability* is $D_i(1-\kappa ) < D_i$ for the same value of $d_i$. Reflecting these changes, we revise Eq. (2), which determines the minimum cash flow level for a bank’s solvency as

$$\begin{aligned} d_i\theta _i \ge&R_c F_i + R(1-\kappa + K\kappa )(1-\kappa ) D_i + \tau (d_i - d_j)^+ = \phi _i +R'd_i + \tau (d_i - d_j)^+ \end{aligned}$$

where $R'=R(1-\kappa + K\kappa )(1-\kappa ) < R$ for a reasonable range of *K* (e.g., $K<2$). Thus, $\mu$ defined in Eq. (8) will decrease to $R'+\tau + R_b/(R + \tau ) -1$. From Eq. (9), we conclude that $R'<R$ lowers bank failure risk. That is, the bank failure risk is reduced by bank capital requirement.

The outcome in the second scenario is different from the findings of Brunnermeier and Niepelt (2019) and Fernández-Villaverde et al. (2020), who argue that a CBDC reduces the likelihood of a bank run. The difference between these results stems from the focus of each study. In previous studies, the transfer of funding from the private sector to the central bank establishes the central bank as a huge liquidity depositor that internalizes the externalities of bank runs, in contrast to small depositors. Meanwhile, in our model, depositors do not run on liquidity or liquidity-convertible assets but rather on less liquid term deposits (or stop rolling over wholesale deposits), which are difficult to convert to or swap with liquidity via monetary policy in their models. The degree of pass-through may be related to the different results of previous studies, which predict no changes (e.g. Brunnermeier and Niepelt 2019; Fernández-Villaverde et al. 2020), overall negative effects (e.g., Keister et al. 2019), and overall positive effects (e.g., Chiu et al. 2019). In a competitive banking environment, a high degree of pass-through of deposit rates to loan markets will occur, and $X_i - R_i$ will consequently decrease. Thus, the adverse outcome is more likely to appear if the reduced liquidity transfer cost ($\tau$) does not affect the bank’s choice of lending ($d_i$), as in Keister et al. (2019). By contrast, our result on lending is close to more positive results, similar to the findings of Chiu et al. (2019) and Andolfatto (2021), where banks have some market power in the deposit market. This helps to absorb the increase in deposit rates caused by CBDC and, consequently, curtail the pass-through of deposit rates to the loan market.

Finally, we note that our results are not derived from the CBDC as a legal tender, but as an innovative payment method compared to conventional ones. Thus, for example, if non-banking institutions can provide an efficient payment method, the market share of the non-banking sector is likely to grow. Therefore, it will create a similar kind of pressure for banks to fund term deposits as does the CBDC, which is a feature also observed in Parlour et al. (2020). Broby (2021) also points that whether the replacement of traditional currency by a CBDC or other digital offering is of secondary importance.

## Discussion and conclusion

We investigate the effects of introducing an economy-wide, account-type CBDC on banks’ loan supplies and bank failure risk using a microeconomic banking model. Similar to many other studies of CBDCs, we regard an account-type CBDC as a strong substitute for conventional demand deposits and reduce the liquidity circulation cost. Our findings are as follows.

First, both the loan supply and bank failure risk increase because banks lower their (excess) liquidity reserves. Simultaneously, CBDC accounts attract liquidity away from bank demand deposits. Consequently, both the amount of banks’ loanable funds and that of their loan supplies will decrease, whereas the rate of return on term deposits and the rate of return on loan, via the pass-through of costs to the loan market, will increase. These two opposing effects ultimately lead to a minor change in loan supply. Unlike the case of loan supply, the ultimate impact on bank failure risk depends on the difference between the rates of return on successful businesses and the rates on loans, which can be interpreted as both the profitability from a successful project, inversely linked to the borrower’s moral hazard, and the favorability of the loan market to the borrowers. If the difference is sufficiently large, implying that the loan market condition is favorable to borrowers, bank failure risk decreases and the ultimate change in the bank failure risk is not significant, as in the case of the loan supply. By contrast, if the difference is small, implying that the loan market condition is less favorable to borrowers, bank failure risk *increases* further, leading to a significant adverse impact. Our findings shed light on some of the seemingly different results of prior studies (for example, Brunnermeier and Niepelt 2019; Chiu et al. 2019; Keister et al. 2019; Fernández-Villaverde et al. 2020; Andolfatto 2021) by specifying the conditions such as profitability of successful businesses and the pass-through of the increase in cost for funds to the rate of return on loans, under which each kind of outcome may be realized.

We emphasize that the bank failure risk refers to that of an individual bank, not a systemic one.^26^ Our findings do not necessarily provide negative policy implications for adopting an economy-wide, account-type CBDC, but rather emphasize the importance of the central bank’s role, which needs to be reinforced in advance to maximize the positive impacts of a CBDC. Specifically, the central bank should help banks minimize their interim liquidity needs and maintain similar gross rates of return to what they were before the introduction of the CBDC. One possible policy approach is to expand the central bank’s short-term liquidity loan facilities (e.g., discount window borrowing from the Federal Reserve System). However, this approach is effective only if little stigma is attached to borrowing, which is not the banking sector’s current sentiment. When a central bank decides whether to implement a token- or account-type CBDC, one must consider the different impacts of the two approaches on bank failure risk as well as operations and regulatory risks. For example, if a central bank adopts a token-type CBDC, non-financial institutions can and should be allowed to provide “wallet” services (e.g., “Novi” by Facebook), which may create various unexpected security problems and complexity in monitoring. Regardless of the medium of implementation, we can conclude that introducing a CBDC would strengthen the central bank’s regulatory role.

We focus on the bank failure risk in this study, but the introduction of a CBDC would influence various aspects of banking. For example, banks’ priority in investment decisions (for example, Kou et al. 2021a) may be altered if an economy-wide CBDC is introduced. Considering that transaction records can be used to estimate the bankruptcy possibility for non-financial businesses (Kou et al. 2021b), the concentration of too much information to central banks would generate concerns about the balance of power among institutions. These topics are beyond the scope of this study, but are worth further investigation in the future.

Lastly, we address limitations of this study. First, the main results of this study are derived from the assumption that the deposit market is competitive, the loan market is monopolistic, and the rates of return are exogenous. However, if the deposit market is regarded as, for example, duopolistic, and the deposit rates are endogenously derived, which causes a significant challenge to the tractability of the problem, and the results may be different. Second, we assume that the ratio of liquidity reserves to the supply of loans is small. Although this assumption is reasonable for many advanced economies,^27^ required reserve ratios are greater than 10%, for example, in some emerging markets. Our results do not apply in such cases. Finally, by assuming that the deposit market is competitive, we implicitly assume that banks earn no profits from demand deposits, which is a condition that plays an important role in determining banks’ liquidity reserves and loan supplies. Although we do not consider these assumptions as excessively unrealistic, our results should be generalized cautiously, given that banks’ non-interest income is related more to checking accounts, which are outside the scope of this study, than to savings accounts. We expect that future studies will address these limitations.

## Appendix 1: Comparative statics

The comparative statics for bank *i*’s chosen (normalized) loan amount, $d_i$ for the major exogenous variables are as follows.

- $\partial d^*_i/\partial B_i=d^*_i/(2B_i)>0$. The number of safer borrowers has increased; thus, bank *i* increases its (normalized) loan amount.
- $$\partial d^*_i/\partial R_c = \frac{\partial d^*_i}{\partial \phi _i}\frac{\partial \phi _i}{\partial R_c}>0$$.
- $\partial d^*_i/\partial R_i = -d^*_i/(2R_i)-d^*_i/(2X_i - 2R_i)<0$. As the gross rate of return from a loan increases, bank *i* reduces its (normalized) loan supply, implying that the expected loss from defaults (i.e., credit risk) becomes greater than the expected gain from the higher rate of return on loans.
- $\partial d^*_i/\partial R = \frac{B_iR_c(X_i - R_i)f_i}{2 \tau R_i}(\tau -R_b/(R+\tau )^2-1)<0$ and $\partial \phi _i / \partial R<0$. As the rate of return on term deposits increases, bank *i* reduces its (normalized) loan amount.
- $\partial d^*_i/\partial \tau = -\frac{d^*_i}{2\tau } + \frac{d^*_i(1- R_b/(R+\tau )^2)}{2(\tau + R_b/(R+\tau )-1)}<0$. As the interbank liquidity transfer cost decreases, bank *i* increases its (normalized) loan amount.
- $\partial d^*_i/\partial f_i = d^*_i/(2f_i)>0$. Bank *i* can and does increase its (normalized) loan supply if it secures a higher level of demand deposits $F_i$, knowing that $f_i = F_i/e$.

The comparative static results for the critical loss level for a risky loan, ${\hat{\gamma _i}}$, are

- $\partial {\hat{\gamma _i}}/\partial B_i={\hat{\gamma _i}} (\frac{1}{B_iR_c} + (-1 + \frac{\phi _i/d_i}{R_i-(\phi _i/d_i + \mu )})\frac{ 1}{2 B_iR_c} )>0$, given that $R_i-(\phi _i/d_i + \mu ) > \phi _i/d_i$. As borrowers become safer, bank *i*’s critical loss level increases, and bank risk falls.
- $\partial {\hat{\gamma _i}}/\partial R_i ={\hat{\gamma _i}}(-\frac{1}{R_i}-\frac{1}{X_i-R_i}+\frac{1}{R_i-(\phi _i/d_i + \mu )}-\frac{1}{d_i}(1-\frac{\phi _i/d_i}{R_i - (\phi _i/d_i + \mu )})\frac{\partial d_i}{\partial R_i} )>0$ if $X_i - R_i$ is sufficiently large. Otherwise, $\partial {\hat{\gamma _i}}/\partial R_i <0$.
  When $R_i$ is sufficiently low, a marginal increase in $R_i$ will increase bank *i*’s profit, which can override the adverse effect from the increased default risk, proxied by small value of $X_i - R_i$. This is due to the increased capital buffer. However, when $R_i$ is sufficiently high, bank *i*’s additional profit from a marginal increase in $R_i$ is not high enough to offset the marginal loss from increased risk of defaults. Thus, the critical loss level decreases, and the bank failure risk increases.
- $\partial {\hat{\gamma _i}}/\partial R = -\frac{{\hat{\gamma _i}}}{d_i} (1-\frac{\phi _i/d_i}{R_i-(\phi _i/d_i + \mu )})\frac{\partial d_i}{\partial R} +\frac{{\hat{\gamma _i}}}{d_i}(\frac{(e-1)f_i/d_i-d_i(1-R_b/(R+\tau )^2)}{R_i-(\phi _i/d_i + \mu )}) > 0$. As the gross rate of return on term deposits increases, the decrease in the bank’s loan supply leads to lower default risk, increases the critical loss level, and, thus, decreases the bank failure risk.
- $\partial {\hat{\gamma _i}}/\partial \tau = -\frac{{\hat{\gamma _i}}}{d_i}\frac{\partial d_i}{\partial \tau }(1-\frac{\phi _i/d_i}{R_i-(\phi _i/d_i + \mu )})-\frac{{\hat{\gamma _i}}}{d_i}\frac{d_i(1-R_b/(R+\tau )^2)}{R_i-(\phi _i/d_i + \mu )} > 0$ because $\frac{\partial d_i}{\partial \tau }<0$ and $1-R_b/(R+\tau )^2<0$. As the cost of inter-bank cash flow transfers decreases, the loan supply increases. Consequently, the critical loss level decreases, and the bank failure risk increases.
- $\partial {\hat{\gamma _i}}/\partial f_i = -\frac{{\hat{\gamma _i}}}{2f_i}-\frac{{\hat{\gamma _i}}}{2f_i} (\frac{\phi _i/d_i}{R_i - (\phi _i + \mu )})<0$. This result appears counterintuitive at first, which should be interpreted as follows. If bank *i* can secure a higher level of demand deposits $F_i$, knowing that $f_i = F_i/e$, it increases its (normalized) loan supply to the point where the critical loss level decreases and the bank failure risk increases.

## Appendix 2: proof of Lemma 1

From Eq. (2), we know the first part of Eq. (6), $\phi _i /d_i + R + \tau < \theta _i$, is satisfied where the cash flow level is immune to insolvency.

The second part of Eq. (6), $\theta _i < \phi _i/d_i + \mu$, is derived from the switching strategy equilibrium of the bank run game, which follows the standard setting of binary action global game satisfying Laplacian property —continuum players, uniform prior, private signal about true state with a small noise, and monotone and continuous payoff. Morris and Shin (2001) demonstrate that a unique optimal switching strategy exists in equilibrium for this kind of games. The threshold signal $s^*$ for depositors’ switching strategy is derived as follows.

In our setting, a depositor is supposed to believe that the likelihood of other depositors’ decision about run (or hold) on a bank behaves like a random variable drawn from the uniform distribution *U*[0, 1].^28^ That is, the fraction of depositors who receive a signal greater than the threshold $s^*$ (i.e., who do not choose early withdrawal, or run), denoted by $M_i$, follows *U*[0, 1].^29^

Equation (5) implies that the proportion of early withdrawals $L_i$ must be lower than $L^*_i$ for bank *i* to survive at $t = 2$. Because the probability of bank failure at $t = 2$ is $\Pr (1 - M_i > L^*_i)$, the ratio of early withdrawals exceeding $L_i^*$, the probability that the bank survives, is given as follows:

$$\begin{aligned} 1 - \Pr (1 - M_i > L^*_i) = 1 - \Pr (M_i < 1 - L^*_i) = 1 - (1 - L^*_i) = L^*_i. \end{aligned}$$

When a depositor is indifferent between early withdrawals and waiting, the equation

$$\begin{aligned} \Pr (\text{ survival } t = 1 | s^i_n=s^*) \cdot d_i = \Pr (\text{ survival } t = 2 | s^i_n=s^*)d_i(R+\tau ) \end{aligned}$$

must be satisfied. $$\Pr ($$survival $$t = 1 | s^i_n=s^*)=1$$, and $$\Pr ($$survival $$t = 2 | s^i_n=s^*)= L_i^*$$ leads to $$d_i = L_id_iR$$. Thus,

15 $$\begin{aligned} d_i = L_i d_i R \le L^*_i d_i R =\frac{\theta _i - (\phi _i/d_i + R + \tau ) }{( R_b - R - \tau ) } d_i (R+\tau ) \end{aligned}$$

should be satisfied if *i* does not face a bank run because $L_i$ follows *U*[0, 1]. After rearranging Eq. (15), we can conclude that bank *i* is illiquid at $t=1$ if the cash flow satisfies the following inequality

$$\begin{aligned} \theta _i \le \phi _i/d_i + R + \tau + (R_b - R - \tau )/(R+\tau ), \end{aligned}$$

implying that $$s^*=\phi _i/d_i + R + \tau + (R_b - R - \tau )/(R+\tau )$$.

## Footnotes

1 The Bank for International Settlements (BIS) provides a comprehensive annual survey of central bank positions and digital currency issuance plans (Barontini and Holden 2019; Boar et al. 2020).

2 Currently, the Fed’s annual currency operating budget is approximately $1 billion ($1,095.8 mil. in 2021). (source: https://www.federalreserve.gov/faqs/currency_12771.htm).

3 Except for several *stable coins* (e.g., Libra) that are backed by other assets, most blockchain-based cryptocurrencies provide no price stability measures, making their use as full-scale currencies impractical.

4 CBDC issuances can be broadly divided into two categories: token and account types (Mersch 2017; Mancini-Griffoli et al. 2018). The token type is similar to an existing cash or gift voucher, as it combines value information with an electronic token and allow the owner to access the value. Conversely, the account type is similar to conventional demand deposits or Bitcoin, as each individual has an account linked to the digital currency, possibly at the central bank (for example, Niepelt 2018). Mancini-Griffoli et al. (2018) provide a more detailed comparison of these two types.

5 Kumhof and Noone (2018) describe three models of the possible extent of CBDC introduction: (i) financial institutions access (Model FI), in which individuals are not allowed to use digital currencies directly; (ii) economy-wide access (Model EW), in which individuals are given direct access to digital currencies; and (iii) financial institutions plus CBDC-backed narrow bank access (Model FI+), a hybrid of the first two.

6 Here, we use the term “demand deposits” in a broad sense. Our definition of this term includes other retail deposits with deposit insurance, such as money market deposit accounts, which are included in the M1 class of money but are classified as savings accounts.

7 Rubinstein (1989), Carlsson and Van Damme (1993) and Morris and Shin (2001) introduce and develop the global game model, which have been applied to various coordination situations, such as (bank) runs, currency attacks, and regime changes. As Goldstein and Pauzner (2005) note, one advantage of the global game approach is that the (analytical) probability of a bank run in equilibrium can be derived.

8 Focusing on monetary policy and prudence regulations for central banks, Raskin and Yermack (2016), Bordo and Levin (2017), Bank for International Settlements (2018), and Kumhof and Noone (2018) provide expository surveys and policy suggestions regarding the effects of CBDC introductions.

9 Kahou and Lehar (2017) provide a summary of financial stability and macro-prudence studies.

10 Our model can be extended to include multiple homogeneous banks in one region, but such an extension provides no additional implications for banks’ failure risks and portfolios.

11 In this study, we use “demand deposits” to refer to non-maturity deposits less prone to bank run, and use “term deposits” to refer not only to deposits with maturities but also to all other deposits more prone to bank runs.

12 By reserve banking, banks use a significant portion of demand deposits as a source for their loan supply, implying that $d_i \ge D_i$ and $f_i \le F_i$.

13 In Freixas and Ma (2014), the fraction of riskless loan is $\alpha \equiv 1 - 1/(B(X - R))$ (no subscript used) because they assume that all entrepreneurs receive loans (i.e., $d_i =1$).

14 We reflect the fact that retail term deposits typically do not carry a penalty, such as a principal loss, for early withdrawals. For wholesale deposits, a more appropriate interpretation is that the wholesale depositors stop rolling over their deposits, such as certificates of deposits, as in Rochet and Vives (2004).

15 This assumption does not mean that no withdrawals occur at all; instead, it means that the average balance of the bank’s demand deposits remains (almost) the same.

16 $R_b$ may range from the gross rate of return in the money market through the gross rate of return on certified deposits up to the gross *discount* rate for *fire sales* (for example, Shleifer and Vishny 2011) of banks’ assets. We regard $R_b$ as an average rate that arises in time of typical stress, rather than as an extraordinarily high value observed in a financial crisis such as the “repo-run” during the 2008 global financial crisis (Gorton and Metrick 2012). That is, the discount rate of the bank’s financial claims applied to its interim sales satisfies $R< R_b < R_i$.

17 Most payment systems, including FedWire and the Clearing House Interbank Payments System (CHIPS), have explicit (albeit small) fees that must be paid for using the system. In addition, many payment systems require banks to pre-deposit collateral, in proportion to its net outflows, to the system before they can participate. For example, in the US, the CHIPS requires the participant institutions to post collateral based on their historical transfer volume before they can participate in the system. Collateral used to participate in the payment system cannot be used for other purposes (e.g., raising funds in the repo market), and so a second opportunity cost arises (Parlour et al. 2020).

18 For example, in Korea, the required reserve ratio of demand deposits is 7% and the proportion of demand deposits is about 15%, implying that a bank retains about 1% of the total deposits as a liquidity reserve, or $f_i \approx 0.01$. The interbank transfer cost $\tau$ varies depending on definition. The cost of operating a large-value payment system that banks collectively need to cover is minuscule compared to the transaction amounts. Still, we need to add opportunity costs for bank deposits to large payments, which Pagès and Humphrey (2005) estimate to be 25 basis points (annual rate), or 0.0025, for the case of CHIPS. In all, we can safely conclude that $\tau < f_i$.

19 The terms and definitions used in this subsection are based on those used by Freixas and Ma (2014).

20 This feature reflects the fact that demand deposits and the CBDC, as opposed to term (and wholesale) deposits, already cover a substantial portion of the actual liquidity demand.

21 According to Laplace, a nineteenth century French mathematician, “One should apply a uniform prior to unknown events “from the principle of insufficient reason”” (re-quote from Morris and Shin 2001, p. 2). The Laplacian property also assumes away the process of opinion or sentiment formation (e.g., Zha et al. 2020) in financial markets.

22 It is noteworthy that both the loan supply $d_i$ and the bank failure risk ${\hat{\gamma _i}}$ are supposed to be endogenously and simultaneously determined by other exogenous factors.

23 Until now, the growing use of electronic means of payment has generally not yet resulted in a substantial reduction in the demand for cash (e.g., Bank for International Settlements 2018).

24 For instance, a CBDC requires neither fees for transfers nor a minimum balance, which is more beneficial for depositors with low initial liquidity endowments.

25 Owing to the uniform distribution assumption on $\gamma _i$, the critical loss level ${\hat{\gamma _i}}$ in our base case, 0.180 or 18% becomes highly unrealistic. We recommend limiting the interpretation of the critical loss level to qualitative implications. We suggest interpreting other values, such as $\phi _i=0.00765$, $\mu = 1.030$, and $\alpha _i=0.926$, similarly.

26 Kou et al. (2019) provide a survey about systemic risks in the financial market with various machine learning methods.

27 For example, the required reserve ratio is 1% in the European Union, 0.8% in Japan, and 0.5% in Canada (source: https://www.ceicdata.com/en/indicator/reserve-requirement-ratio).
